# Mandibular Fracture in a Child Resulting from a Dog Attack: A Case Report

**DOI:** 10.1155/2011/659756

**Published:** 2011-07-19

**Authors:** Hannah Cottom, Dery Tuopar, Phillip Ameerally

**Affiliations:** Department of Oral and Maxillofacial Surgery, Northampton General Hospital, Northampton NN1 5BD, UK

## Abstract

Dog attacks are extremely frequent and are thought to be responsible for an average of 250,000 minor injuries and emergency unit attendances each year. Children in particular are more likely to experience dog-bite injuries with 5–9-year olds most susceptible. The majority of injuries are to the head region, with the lips, cheeks, and nose often affected. Most injuries experienced are confined to the soft tissues; nevertheless, maxillofacial fracture is a potential albeit rare complication. The incidence of facial fractures in relation to dog bites is unknown; however, some have estimated that facial fractures could occur in 5% of dog attacks. However mandibular fracture following a dog bite is extremely rare, with review of the literature only identifying three cases. We present a further case in which a five-year-old sustained numerous soft-tissue lacerations to the face and hand, together with fracture of the mandibular symphysis following a dog attack. The fracture was successfully repaired using open reduction and internal fixation with titanium plates and screws. The case emphasises that although maxillofacial fracture is rare, it may occur following a dog bite and that thorough and systematic examination of the facial skeleton is crucial to exclude the presence of such injuries.

## 1. Introduction

In the UK, it is estimated that dog attack injuries are responsible for an average of 250,000 minor injuries and emergency unit attendances each year [1]. The incidence of dog-bite injuries in children below the age of fifteen is thought to be 22 in 1000 every year [2]. Similar high incidences have also been reported by Karlson [3] and Shaikh and Worrall[4], who documented dog bites as the cause of facial injury in 27.4% of children under the age of seven years old [3, 4]. The reason for these high occurrences in young children has been attributed to lack of awareness of the potential danger from animals and the closer proximity children have to dogs as a result of their stature [4]. 

In children, over three-quarter of dog attacks are to the face and head [5–9]. The most common sites affected are the lips, ears, cheeks, and nose [10–12]. Predominantly, the injuries encountered are restricted to the soft tissues and are designated into three categories: lacerations, punctures, and avulsions (tissue loss). The resulting soft-tissue injuries can additionally vary considerably in relation to their extent and depth [13]. Maxillofacial fractures are uncommon in young children [4, 13, 14], and consequently, bone fractures are often not even considered following dog attack injuries to the face. The actual incidence of facial fractures relating to dog attacks is currently unknown. Schalamon et al. [14], Karlson [3], and Palmer and Rees [9] documented no maxillofacial fractures in their review of facial dog-bite injuries, and Tu et al. [13] suggested that facial fractures may occur in less than 5% of dog attack incidents [3, 9, 13, 14]. When a maxillofacial fracture is encountered, the most frequent bones to be fractured are the orbital, nasal, and maxillary bones, constituting 78% of the documented dog-bite facial fractures [13, 15]. Less commonly reported fractures include the zygoma, skull, and the mandible. Extensive review of the literature has revealed only three cases, in which the mandible was fractured as a consequence of a dog attack [1, 16, 17]. In all three cases the child involved was below the age of five, and the fracture affected the body or angle of the mandible.

We present an interesting case in which a five-year-old child presented with multiple soft-tissue lacerations to the face and hand together with a fractured mandible in the symphysis region, all sustained from a dog-bite attack.

## 2. Case Report

**Figure 1:**
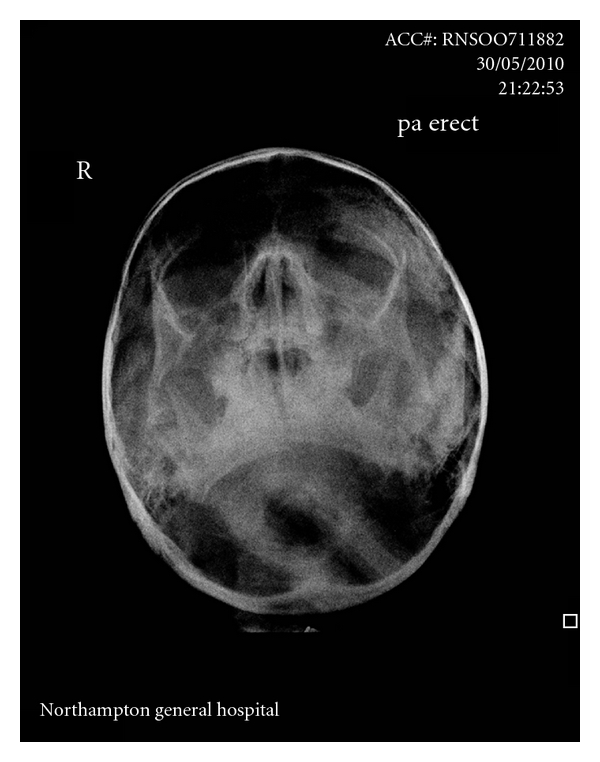
Postero-anterior view revealing a displaced fracture to the mandibular symphysis.

A five-year-old girl presented following an attack by the family dog. She was otherwise fit and well and had no relevant medical history or known allergies. Examination revealed multiple soft-tissue lacerations to the face which comprised of a 10 cm deep laceration over the right lower border of the mandible down to the bone (sustained by the dog biting and gripping the mandible with its teeth), a 3 cm laceration above the left eyebrow, multiple small superficial lacerations to the left cheek, a 4 cm laceration to the left cheek through to the parotid capsule, a 4 cm laceration present on the left nasolabial fold through to the mouth, and a ragged laceration over the bridge of the nose. A laceration was also present on the right hand on the palmar aspect of the second web space, approximately 1 cm in length. Intraoral examination and assessment of the facial nerve was not possible due to agitation and distress experienced by the patient. Nevertheless, facial radiographic imaging was performed and postero-anterior views revealed fracture of the mandibular symphysis (Figure 1). 

**Figure 2:**
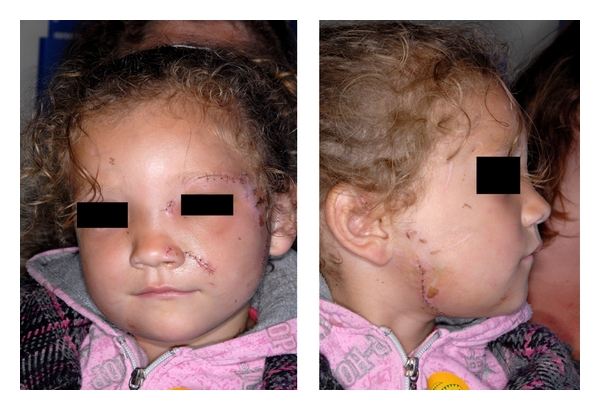
The extent of the soft-tissue injuries sustained from the dog-bite attack.

Following hospital admission, the patient was taken to the operating theatre. Her soft-tissue wounds were thoroughly debrided and irrigated with normal saline and sutured. In addition, the bilateral deep cheek lacerations were surgically explored, and the facial nerve branches were found to be intact. An EUA also revealed a luxated upper left deciduous central incisor with an associated upper labial gingival laceration. A degloved laceration was additionally noted in the lower right buccal sulcus adjacent to the second deciduous molar and first permanent molar. The mandible was found to be mobile in the symphysis region, and there was an obvious displaced fracture. This was reduced and fixed with two 1.3 mm titanium plates. The upper left deciduous central incisor was extracted and the lower right buccal sulcus laceration sutured. The plates were removed after 6 weeks, and it was noted that there was solid bony union at the previous fracture site. The child was reviewed and finally discharged.  Figure 2 demonstrates the extent of the soft-tissue injuries sustained.

## 3. Discussion

Dog-bite injuries are extremely frequent, and in the USA, an average of 4.7 million dog bites occur each year with approximately 799,700 people needing medical care as a result [18]. Children in particular are more likely to experience dog-bite injuries compared to adults, with children aged between 5 and 9 years considered to be most at risk [1, 19]. Therefore, a considerable proportion of facial trauma in children results from dog-bite attacks and represents a significant medical and public health issue [2–4, 14]. 

In the majority of dog attacks, the animal is known to the child and certainly in our case, the dog in question was a family pet [16]. Most dog-bite injuries in children are to the extremities of the body with the face and head stated as the common areas involved [1, 9, 14]. The child in our case received injuries solely to these areas and would support the literature in that the lip and cheeks are affected predominantly [9]. In the vast number of dog-bite injures affecting children, the trauma sustained only involves the soft tissues; however, in very rare cases, facial bone fracture can be experienced. Brogan et al. [15] in their case series revealed that a quarter of reviewed severe dog-bite attacks to the head region resulted in fracture of the skull or facial bones. The vast majority of such maxillofacial fractures are to the orbital, nasal, and maxillary bones [13, 15]. We found only three cases of fracture of the mandible from dog attacks [1, 16, 17]. The mechanism of injury in cases of maxillofacial fracture is thought to be the consequence of the mandible (or involved bone) being physically held by the dogs jaws, which is capable of delivering immense force to the area of bone contacted by the dogs teeth. In some breeds of dog, the force produced has been measured to be in the region of 31790 KPa [1, 20, 21]. The resultant force generated creates a crush-type injury and fracture of the alveolar bone. Young children are especially vulnerable to this type of crush injury, since the maxillofacial skeleton is not completely mineralised, is thinner, and, therefore, considerably weaker compared to during adulthood [13].

The technique utilised to repair the fractured mandibular symphysis was that of the conventional approach of open reduction and internal fixation with titanium plates and screws. The titanium plates stabilised the fracture site and were subsequently removed after six weeks once bony union had been established. The plates were removed in order to minimise the risk of interference with normal growth of the mandible and damaging or disturbing the permanent dentition developing in the alveolar bone. 

Wound infection is the most common complication following these injuries. Some authors estimate an infection rate of up to 30% following animal bite injuries to the extremities [22, 23]. Other complications of bite injuries include hypertrophic scarring; fortunately, this did not occur in our patient. 

This paper emphasises that although maxillofacial fractures resulting from of dog-bite injuries are extremely infrequent, they are, nevertheless, a potential complication, especially in young children. It is, therefore, of paramount importance to perform a systematic examination of the facial skeleton in order to actively exclude the presence of such fractures in patients presenting with facial dog-bite injuries.
